# Leucocytosis after Operations

**Published:** 1906-07-28

**Authors:** 


### LEUCOCYTOSIS AFTER OPERATIONS.

A very valuable research has been carried out in Edinburgh by F. L. Dawson on the condition of the blood after operations and fractures. Some 50 operation cases were examined with a view to find out whether a routine blood-examination would give useful information when the patient does not appear to be doing well. One remarkable result met with was that after all operations a considerable leucocytosis occurs, reaching a maximum some hours after operation, which is chiefly due to an increase of the polymorphonuclears. There is some increase of the large mononuclears, but a decrease in the small lymphocytes and eosinophiles. Sometimes, also, a glycogen reaction is present.[1] This leucocytosis is not due to hæmorrhage, the anæsthetics employed, the frequent slight rise of temperature, or the preparation of the patient, or to surgical methods or the presence of blood-clot or bruised tissues, and it does not occur after simple fractures. The cause of this reaction is not clear, though it is possibly a protective reaction against the few micro-organisms which have entered during the operation. If, however, the leucocytes do not decrease by the second day, or if after the fall a secondary rise takes place, then the wound is probably septic. If the leucocytosis and the polynuclears are decreasing regularly and there is no glycogen reaction, we can be pretty sure that the wound has not gone wrong, even though some pyrexia exists. It is evident that to be able to rely upon the evidence given by this form of leucocytosis, we should make a count before the operation. When this has been done the subsequent course of the reaction seems capable of affording very valuable information as to progress without the need of opening up the wound. Grünbaum[2] gives further indications as to the presence of pus. If we repeat the count four hours later, after the patient's condition has become suspicious, and find that the number of white cells is still rising and that the polynuclears have reached 80 per cent., then the presence of pus is almost certain. We must, however, be able to exclude the presence of pneumonia and small superficial abscesses, such as those around stitches. A confirmatory indication is the iodophilic reaction obtained by painting a film with mucilage containing 1 per cent. of iodine and 3 per cent. of iodide of potash, which gives brownish granules in a few cells, if pus is present and pertussis and pneumonia are absent. The increase of platelets and of fibrin in the blood is additional evidence in favour of pus, and a sign not found in leucocytosis due to malignant growths. The importance of these points when deep-seated abscesses or suppuration are feared is very great, but it should be remembered that abscesses due to the amœba coli or the tubercle bacillus may exist without giving these symptoms.

[1] Scottish Med. and Sur. Jour., Nov. [2] Practit. Dec.

